# Travelers as Sentinels for Chikungunya Fever, Brazil

**DOI:** 10.3201/eid1803.110838

**Published:** 2012-03

**Authors:** Tânia do Socorro Souza Chaves, Alessandra Cristina Guedes Pellini, Melissa Mascheretti, Maria Teresa Jahnel, Ana Freitas Ribeiro, Sueli Guerreiro Rodrigues, Pedro Fernando da Costa Vasconcelos, Marcos Boulos

**Affiliations:** University of São Paulo School of Medicine, São Paulo, Brazil (T.S.S. Chaves, M. Boulos);; Emílio Ribas Institute of Infectious Diseases, São Paulo (T.S.S. Chaves);; Center for Epidemiological Surveillance, São Paulo (A.C.G. Pellini, M. Mascheretti, M.T. Jahnel, A.F. Ribeiro);; Evandro Chagas Institute, Belém, Brazil (S.G. Rodrigues, P.F.C. Vasconcelos)

**Keywords:** chikungunya virus, travel, Aedes mosquitoes, vector-borne diseases, emerging communicable diseases, public health, epidemiologic surveillance, Brazil

**To the Editor:** The reemergence of chikungunya virus (CHIKV) infection recently has been reported in travelers after they returned from affected areas (*1**–**6*). In the Americas, local transmission has not been identified, although imported cases have been reported in travelers returning from Reunion Island to Martinique, French Guiana, and Guadeloupe (*7*). In the United States, CHIKV infections have also been reported in travelers who returned from disease-endemic areas (*8*).

Climate changes in recent decades have affected the dynamics of infectious disease transmission, increasing the incidence, prevalence, and number of outbreaks of mosquito-borne diseases, such as dengue fever. Both CHIKV and dengue virus are transmitted by *Aedes* spp. mosquitoes. *Ae. aegypti* mosquitoes are the most common mosquito involved in dengue transmission, and *Ae. albopictus* mosquitoes have been described as efficient vectors of CHIKV. Recent global expansion of *Ae. albopictus* mosquitoes has been associated with the introduction and dissemination of CHIKV in new areas (*9*).

More than 4,000 cities in Brazil are infested with *Ae. aegypti* mosquitoes, which predominates in urban areas, and such areas have a high incidence of dengue fever and annual outbreaks of this disease. *Ae. albopictus* mosquitoes have been identified in Brazil, where they are more frequently found in rural areas (*10*). The confirmed chikungunya fever cases described here illustrate the risk for introduction and sustained transmission of the disease in Brazil.

In August 2010, a 55-year-old man returned to Brazil from Indonesia, where he had spent 15 days. Seven days after his arrival in Indonesia, a fever (temperature 38.5–39.0°C) developed that lasted for 3 days, along with a facial rash that spread to his neck, trunk, legs, and ankles, followed by desquamation. During the trip, he experienced disabling pain and swelling in the ankles, accompanied by weight loss (5 kg). Four other travelers in his group experienced fever, arthralgia, and malaise. Upon his return to Brazil, the man immediately sought medical attention, and his symptoms were treated with intravenous fluids, parenteral corticosteroids, and nonsteroidal antiinflammatory drugs for 2 weeks. Despite improvement, the arthralgia recurred in the wrists and metacarpal bones. He was referred to the Travel Medicine Outpatient Clinic of the University of São Paulo School of Medicine Hospital das Clínicas. Laboratory tests showed elevated levels of aspartate transaminase (117 U/L), alanine transaminase (179 U/L), and C-reactive protein (27.8 mg/L). Test results for *Plasmodium* spp., dengue virus, cytomegalovirus, and *Toxoplasma* spp. were all negative. Fifty-three days after onset symptom, anti-CHIKV IgM and IgG antibodies were detected by ELISA. By day 60, his IgG titer had risen from 3,200 to 6,400, where it remained 11 months after onset of symptoms.

In October 2010, a 25-year-old woman returned to Brazil from Rajasthan, India, where she had spent 30 days working with a humanitarian aid group. During her return, fever (38.0–39.0°C) and malaise developed. She sought medical attention in the emergency department of the Emílio Ribas Institute of Infectious Diseases, reporting fever, headache, myalgia, fatigue, general malaise, and paresthesia of the hands, as well as severe ankle and foot pain with gait impairment. Physical examination showed dehydration, conjunctival injection, and fever (temperature 38.0°C), as well as skin redness and a faint rash on the trunk. She also had swollen ankles. The fever (temperature 37.8°C) persisted, and she had pain in her ankles and left knee, which made it difficult for her to walk, accompanied by desquamation of palms and soles (Figure). Laboratory tests detected leukopenia and thrombocytopenia. Test results for *Plasmodium* spp. and dengue virus were negative, and blood culture results were negative as well. By using ELISA, anti-CHIKV IgM antibodies were detected 10 days after onset of symptoms, and anti-CHIKV IgG antibodies (titer 25,600) were detected 8 months later.

**Figure:**
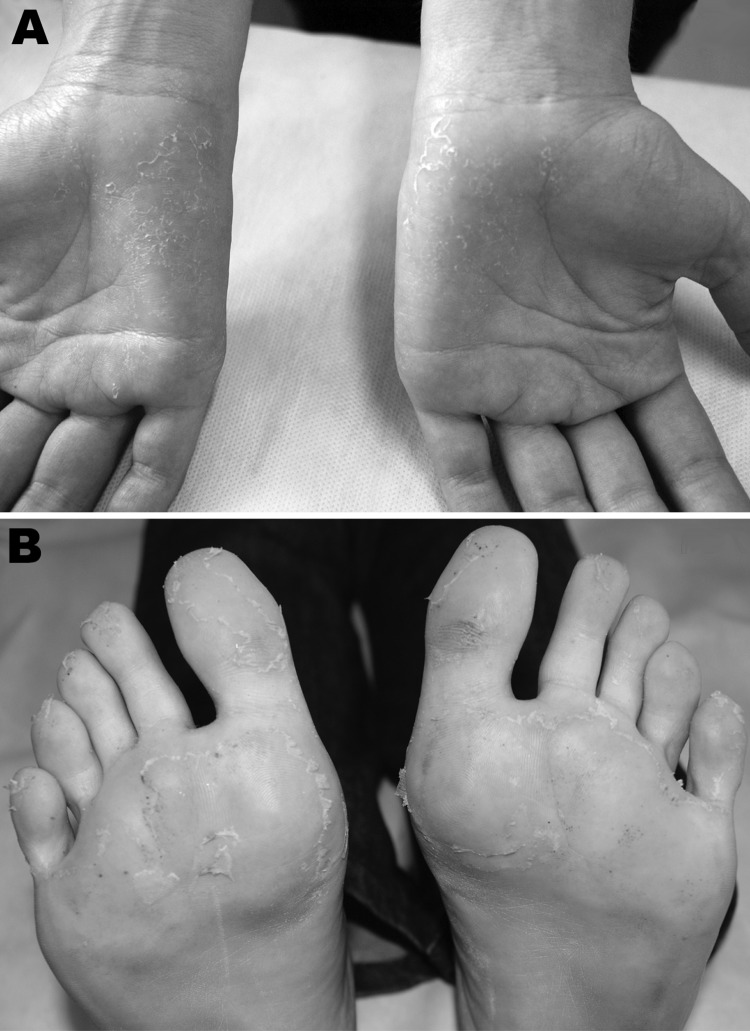
Clinical features exhibited by patient with chikungunya, Brazil 2010. A) Desquamation of palms after maculopapular rash, 33 days after symptom onset. B) Desquamation of soles after maculopapular rash, 33 days after symptom onset.

Both patients were diagnosed after the viremic period; no virus could be isolated or genotyped. Nevertheless, health authorities were alerted and appropriate control measures were taken.

Travelers can serve as sentinels for the introduction of viruses into previously non–disease-endemic areas. Several reports have been made of travelers carrying CHIKV to and from many regions of the world (*2**,**4**–**6*). Recent identification of the expansion of infested areas by *Ae. aegypti* and *Ae. albopictus* mosquitoes, population susceptibility for the virus, and the constant journeying of travelers from affected areas are relevant indications of the risk for introduction and sustained transmission of CHIKV in Brazil.

Health care professionals and public health authorities should be aware of the epidemiologic and clinical aspects of CHIKV infection and diagnoses to adopt prompt control measures to avoid CHIKV transmission in Brazil. Healthcare facilities and epidemiologic surveillance teams have jointly implemented CHIKV prevention and control measures. To date, no autochthonous transmission of CHIKV has been reported in Brazil.
